# Detection, persistence, and rising prevalence of oncogenic viruses revealed by wastewater metagenomics

**DOI:** 10.1128/aem.00547-26

**Published:** 2026-05-13

**Authors:** Harihara Prakash, Ryan K. Perez, Matt Ross, Michael Tisza, Sara J. Javornik Cregeen, Jennifer Deegan, Joseph F. Petrosino, Eric Boerwinkle, Justin R. Clark, Anthony W. Maresso

**Affiliations:** 1 Department of Molecular Virology and Microbiology, Baylor College of Medicine 3989, Houston, Texas, USA; 2 TAILФR Labs, Baylor College of Medicine 3989, Houston, Texas, USA; 3 Alkek Center for Metagenomics and Microbiome Research, Baylor College of Medicine 3989, Houston, Texas, USA; 4 The University of Texas Health Science Center at Houston (UTHealth) School of Public Health 49219, Houston, Texas, USA; Centers for Disease Control and Prevention, Atlanta, Georgia, USA

**Keywords:** oncogenic virus, wastewater based epidemiology, hybrid-capture sequencing, cancer

## Abstract

Oncogenic viruses cause high-risk cancers in humans and are responsible for nearly 20% of all cancer cases worldwide. Currently, very limited data exist in the realm of wastewater-based viral epidemiology (WBE) for cancer-causing viruses, with existing studies using targeted approaches (i.e., PCR-based approaches) that lack genomic resolution. In this study, we used a hybrid-capture approach to detect, filter, and sequence all known oncogenic virus signals from wastewater samples collected over 3 years (May 2022–May 2025) in 16 Texas cities, covering nearly 25% of the state’s population. Once sequenced, we used custom computational tools designed for wastewater metagenomics to assign reads into their respective virus of origin, estimate viral abundances over time, and measure genomic read coverage. Our data indicate that we successfully detected oncogenic viruses, including six known oncogenic viruses, and three suspected oncogenic viruses, across all sampling locations within Texas. We observed a gradual increase in the viral abundance of oncogenic viruses over 3 years, with distinct peaks and dips over the summer and winter months. The prevalence of high-risk viruses such as human papillomavirus (HPV) and Epstein-Barr virus (EBV) rose, with sharp increases in viral abundance observed post-2024. We also obtained nearly 100% genome coverage with viral reads captured using this hybrid-capture technique for nearly all oncogenic viruses, with resolution down to the species and type taxonomic levels in some cases, such as that of HPV. Our study showcases the utility of hybrid-capture techniques to detect and track multiple oncogenic viruses simultaneously.

**IMPORTANCE:**

Cancer-causing viruses are of major clinical significance, responsible for nearly 20% of all recorded cancer incidences in humans worldwide. There is a need for improved detection, tracking, and control of oncogenic viruses across the globe. To our knowledge, this work is the first comprehensive WBE approach used to detect all known oncogenic viruses concurrently, demonstrating the feasibility of monitoring the presence and levels of cancer-causing viruses and enabling the possibility of public health interventions in the future. Using this method, we obtain broad genomic coverage at strong depth and specificity, coupled with consistent real-time tracking dynamics of multiple oncogenic viruses. Furthermore, we showcase the ability to identify genomic regions on viral reference genomes from which sequenced reads originate. This information can be an invaluable tool toward understanding the viral prevalence dynamics in general populations, their relationship to cancer incidences in humans, and their mechanisms of viral evolution, including mutations.

## INTRODUCTION

Oncogenic viruses are responsible for 20% of all human cancers (1). These viruses span at least six distinct viral families and drive cancer formation through a variety of diverse mechanisms (1, 2). They have been linked to cancers of the cervix, anus, vagina, penis, oropharynx, liver, and other sites, with broad clinical implications (3–6). Among the known oncogenic viruses are hepatitis B virus (HBV), hepatitis C virus (HCV), high-risk human papillomaviruses (HPV), Epstein-Barr virus (EBV), human T-cell lymphotropic virus (HTLV-1), and Kaposi sarcoma herpesvirus (KSHV). These viruses are classified as Group 1 carcinogens by the International Agency for Research on Cancer (IARC). Others, like Merkel cell polyomavirus (MCPyV) and BK polyomavirus (BKV), along with other human polyomaviruses (HPyV), are strongly linked to cancer and are known carcinogens (7, 8).

Because these viruses often establish chronic, persistent infections, symptoms may not appear until years or decades after exposure. Cervical cancer caused by high-risk HPV, for example, develops slowly over time, while hepatocellular carcinoma (HCC) resulting from chronic HBV infections is not seen until middle age or later (9, 10). This long latency period complicates public health efforts to interrupt transmission and intervene before cancer develops. Since most afflicted individuals are unaware of infection until physical symptoms manifest much later in life, tracking infections for public health interventions becomes difficult. Even then, infected immunocompromised individuals experience symptoms much sooner than immunocompetent individuals following infection by an oncogenic virus (11). These factors make correlation of present-day clinical cases with present-day viral prevalence tricky, primarily due to the relatively late onset of life-threatening symptoms.

HPV is the most common viral sexually transmitted infection (STI), causing nearly 95% of all cervical cancer in women worldwide, with over 42 million infections recorded in 2018 in the United States alone (5, 6, 12). Among the nearly 200 known HPV genotypes, HPV16 and 18, members of the alpha-papillomavirus genus, are responsible for over 70% of all recorded cervical cancer cases (5, 13, 14). HPV genera are classified into alpha-papillomavirus, beta-papillomavirus, gamma-papillomavirus, mu-papillomavirus, and nu-papillomavirus based on the L1 capsid protein, with further clinical classification of genus-specific genotypes into high- and low-risk based on their ability to cause life-threatening or benign tumors, respectively (15, 16). Clinically relevant members of the alpha-papillomaviruses genus are sub-classified into 14 known high-risk genotypes and 11 low-risk genotypes (17). In addition to this classification, these HPV genera differ in their target organ within the human body, with the alpha-HPV genotypes infecting mucous membranes, while the beta- and gamma-HPV genera primarily cause benign cutaneous lesions and skin cancers, respectively (18, 19). The clinically relevant oncogenic viruses are not limited to HPV, with multiple hepatitis viruses also responsible for causing cancer in humans. HBV and HCV, also known as STIs, are the primary causative agents of HCC, responsible for an overwhelming 80% of all cases (1 million cases by the end of 2025) (4, 20). Recently, the hepatitis D virus (HDV) has also been included as a known human carcinogen by the IARC, and while not oncogenic by itself due to its biology, it co-infects a human host with HBV, accelerating the development of HCC in afflicted individuals and significantly weakening their immune systems (21–23).

Wastewater-based viral epidemiology (WBE) is a promising public health surveillance approach to monitor viral infections at the population level (24). WBE emerged as a valuable, non-invasive tool during the SARS-CoV-2 pandemic, with countries across the Americas, Europe, Asia, and Oceania utilizing this approach to assess community health (25). A prime advantage of WBE is its ability to facilitate the assessment of community health without requiring active clinical testing of individuals. Due to the pooled nature of wastewater sampling and testing, single-patient anonymity is preserved, preventing stigmatization of communities or neighborhoods. WBE also helps public health departments understand disease dynamics in underdeveloped regions, where factors like poverty, poor responses to clinical testing, social stigma associated with disease, and more contribute to underreporting of clinical cases. This is particularly useful in assessing the prevalence of oncogenic viruses, where individuals are completely unaware of infection and do not report it until much later when life-threatening symptoms begin to manifest. While WBE cannot directly notify an individual of viral infection, it can serve as a proxy for assessing community-wide public health, informing officials, and letting them take appropriate actions.

Popular targeted bioanalytical methods used in WBE include liquid chromatography-tandem mass spectrometry (LC-MS/MS) and quantitative polymerase chain reaction (qPCR), which help researchers track the abundance of medically relevant bacteria, viruses, and biomarkers in wastewater (26, 27). Currently, WBE has been used to detect the presence of high-risk oncogenic viruses such as HPV, HBV, and HCV in countries such as Canada, the USA, and China, where targeted PCR and biomarker-based LC-MS/MS approaches were used to measure the prevalence of the viruses across regions (27–29).

The Texas Epidemic Public Health Institute (TEPHI) launched the TexWeb initiative in May 2022 to track viral outbreaks with a metagenomic sequencing-based approach (30). Instead of targeting individual species via multiplexing PCR, TexWEB uses a custom hybrid-capture probe set to capture over 3,000 human and animal viruses, including high-impact viruses such as mpox, SARS-CoV-2, influenza, dengue, avian flu, and more (24, 31–34). As of 2025, this system routinely samples and analyzes wastewater from more than 40 sites in 16 cities across Texas, monitoring over 500 different viruses and reporting over 15,000 different viral variants (30).

In our study, we utilize this approach to track multiple oncogenic viruses across Texas, showcasing the capacity of hybrid-capture sequencing in complementing targeted WBE approaches. While we detected all known oncogenic viruses in wastewater sampled across the state with this method, we also chose to look deeper into three clinically relevant viruses that have been medically established to cause high-risk cancers in humans, namely HBV, HCV, and HPV. We aimed to identify key genomic regions from which sequencing reads of these viruses originate while also assessing the taxonomic depth to which sequencing reads could be classified. In addition, although hepatitis A virus (HAV) and hepatitis E virus (HEV) are not oncogenic viruses, we briefly include them to provide context for interpreting hepatitis viral dynamics more broadly (35). Our results show high read depth and broad genome coverage across multiple viruses, including the ability to track HPV diversity at the genus level. We also find the prevalence of multiple known oncogenic viruses increasing over a span of 3 years of consistent sampling. Our work demonstrates how hybrid-capture sequencing enables both robust detection, as well as genomic characterization of oncogenic viruses in wastewater. These findings support the use of sequencing-based WBE as a surveillance tool for cancer-associated viruses and lay the groundwork for future efforts that link wastewater signals to clinical outcomes and targeted interventions.

## RESULTS

### Oncogenic viruses are consistently detected in wastewater across Texas

To evaluate the presence of oncogenic viruses in wastewater samples and validate the utility of hybrid-capture sequencing, we analyzed samples collected by TexWeb over a 3-year period (May 2022–May 2025). This included over 3,000 distinct samples with a geographic range between the most distant cities of 750 miles. Oncogenic viruses were consistently detected across this time frame, with ongoing monitoring at urban catchment sites throughout the state. When viral read counts were aggregated across all sampling sites, all nine oncogenic viruses (both known and suspected) were detected. To quantify viral abundance, we used RPKMF (calculated as Reads Per Kilobase of reference genome/Million reads passing Filtering) as our abundance metric. RPKMF is a relative abundance metric, dependent on read length and sequencing depth, and does not represent absolute levels of viral load. However, we utilized this relative metric to assess the spatial and temporal patterns of oncogenic viruses in Texas, allowing comparisons of viral prevalence relative to other entities in the collected samples. To verify if relative abundance can be used as a valid proxy for absolute abundance of viral load, we standardized samples with an SV40 spike-in control. After normalizing the viral RPKMF of oncogenic viruses by the known volume of SV40 spike-in, we observed similar prevalence trends between raw and normalized viral levels across all oncogenic viruses (Fig. S3), validating that the overall temporal trend was conserved.

**Fig 1:**
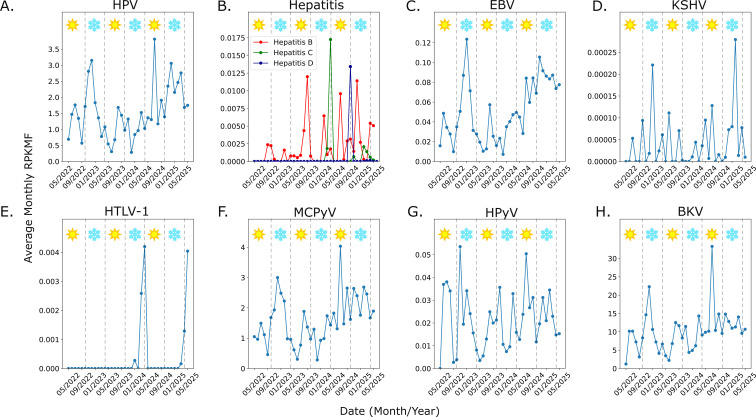
(**A–H**) Abundance of oncogenic virus reads over 3 years of sampling across Texas. Mean viral signal measured in average monthly RPKMF over time in samples taken from wastewater and measured using hybrid-capture probes. Warmer months are highlighted with a sun icon (April–September). Colder months are highlighted with a snowflake icon (October–March). Seasonal boundaries were defined using statewide average temperatures to determine the midpoints between the summer and winter temperature extremes. Both oncogenic and non-oncogenic HPV types are included as one in panel A. HPV, human papillomavirus; EBV, Epstein-Barr virus; HTLV-1, human T-cell lymphotropic virus type 1; KSHV, Kaposi sarcoma herpesvirus; MCPyV, Merkel cell polyomavirus; HPyV, human polyomavirus (specifically alpha- and delta-polyomavirus); BKV, BK polyomavirus.

While site-specific abundance information per virus was recorded, we chose to aggregate read counts monthly by calculating the average monthly RPKMF across all sites taken together. This allowed the observation of viral trends from a broader state-wide perspective, while attempting to discern a possible seasonal prevalence pattern. Thus, taken monthly, we found that the average RPKMF values varied across species, space, and over time, with HPV, MCPyV, EBV, and BKV showing an upward trend over 3 years (Fig. 1).

Some viruses, such as HPV (both oncogenic and non-oncogenic types), EBV, MCPyV, and BKV (Fig. 1A, C, F, and H), were detected in at least one site every month, suggestive of widespread presence. In contrast, others, including HCV, KSHV, and HTLV-1 (Fig. 1B, D, and E), exhibited more frequent non-detection or low read abundance. Despite this, these low-abundance viruses were still detected intermittently across multiple sites, demonstrating the method’s capacity to capture even sparse signals. In contrast, HPyV, specifically alpha- and delta-polyomaviruses (Fig. 1G), did not show any distinct seasonal pattern.

Among the oncogenic hepatitis viruses, HBV and HCV were detected in low amounts (Fig. 1B), with HCV detections very sparse across all sites. HDV was also observed in very low amounts and was also very sparsely detected across the tested sites. To get a full picture of hepatitis transmission dynamics across Texas over 3 years, we examined all hepatitis viruses (Fig. S1). HAV and HEV detections were also sporadic, with no clear pattern, possibly a sign of localized high-intensity outbreaks.

### Genomic coverage analysis of wastewater signals

Building on the above observations, we next analyzed genome coverage for six clinically significant viruses—hepatitis A, B, C, D, E, and HPV—to assess read depth and completeness of genome recovery from hybrid-capture sequencing. These viruses were selected for a deeper genomic analysis based on their medical relevance, and while hepatitis A, D, and E are not oncogenic by themselves, they were briefly included to highlight the viral read dynamics of all hepatitis viruses completely.

**Fig 2:**
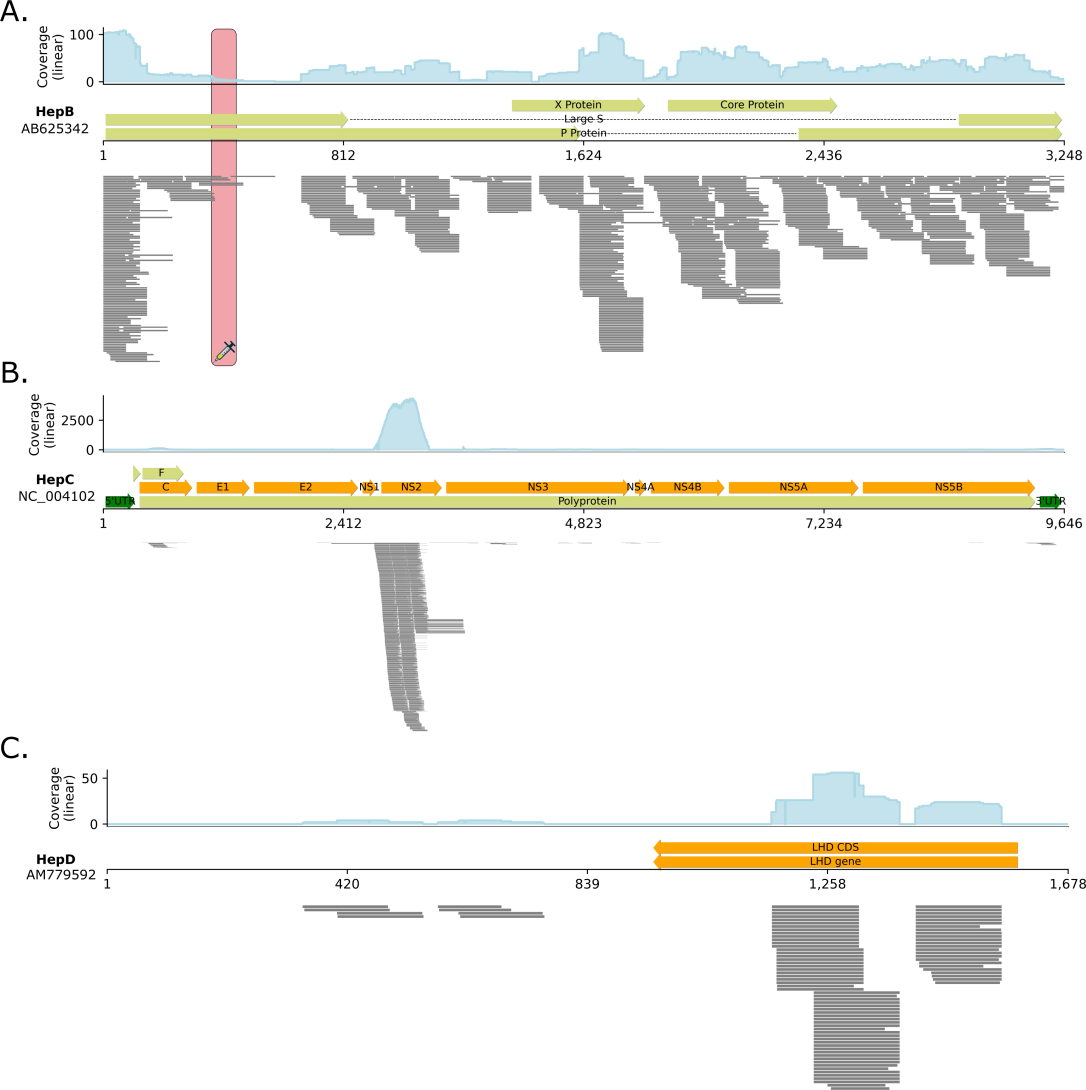
Genome-wide read coverage of sampled hepatitis viruses via hybrid-capture sequencing. The histogram (top) shows coverage depth across the complete genome. Genomic annotations across the genome are highlighted with arrows (middle). Read distribution shown by stacked reads (bottom). Vaccine target regions on the relevant hepatitis genomes are highlighted in red with a syringe icon. (**A**) Reads mapped to the reference hepatitis B genome (accession no. AB625342). (**B**) Reads mapped to the reference hepatitis C genome (accession no. NC_004102). (**C**) Reads mapped to the reference hepatitis D genome (accession no. AM779592).

We mapped hepatitis-assigned reads to all EsViritu Virus Pathogen Database hepatitis references using a multimapping alignment strategy and chose the reference with the most mapped reads (36). In this competitive mapping step, reads that map equally well to multiple references are assigned to all mapped references. Relatively fewer reads were recovered for HBV, with a total of 771 HBV classified reads. Of these, 765 mapped to the selected HBV reference genome (accession no. AB625342), with read coverage observed across multiple functional regions. Despite the lower total count, reads aligned consistently to key genes, including the S (surface) gene, P (polymerase) gene, C (core) gene, and X gene regions, indicating meaningful genomic information can still be recovered from low-abundance targets (Fig. 2A).

For HCV, however, 10,728 out of 10,862 classified reads were mapped to the chosen HCV reference genome (accession no. NC_004102), but coverage was more fragmented. Reads primarily aligned to the core protein, NS2, and NS3 regions, with a disproportionate number mapping to the NS2 region (Fig. 2B). To assess possible vector contamination, we sampled mapped reads and compared BLAST (Basic Local Alignment Search Tool) hits from the Core nucleotide database, with and without restricting to “synthetic construct” sequences (taxid: 32,630) (37, 38). Non-synthetic hits greatly outnumbered synthetic hits, and synthetic hits were for vectors specifically designed to express HCV genes, suggesting vector contamination is unlikely.

HDV had the fewest reads. Of the 96 HDV reads obtained, 88 reads were found to map to the reference HDV genome (accession no. AM779592), with nearly 90% of reads mapping exclusively to the LHD gene region (Fig. 2C).

For the non-oncogenic HAV, all 93,846 reads originally classified mapped to the selected HAV reference (accession no. LC373510) with near-complete coverage and high depth. Read density was strongest across the VP1, VP2, and VP3 regions and extended into other coding and non-coding regions (Fig. S2A). For HEV, 2,341 of 2,847 reads mapped to the selected HEV reference (accession no. LC055972), and coverage was sparse across the genome with a distinct peak in the capsid protein CDS (Fig. S2B).

### HPV genera show distinct prevalence patterns in wastewater

**Fig 3:**
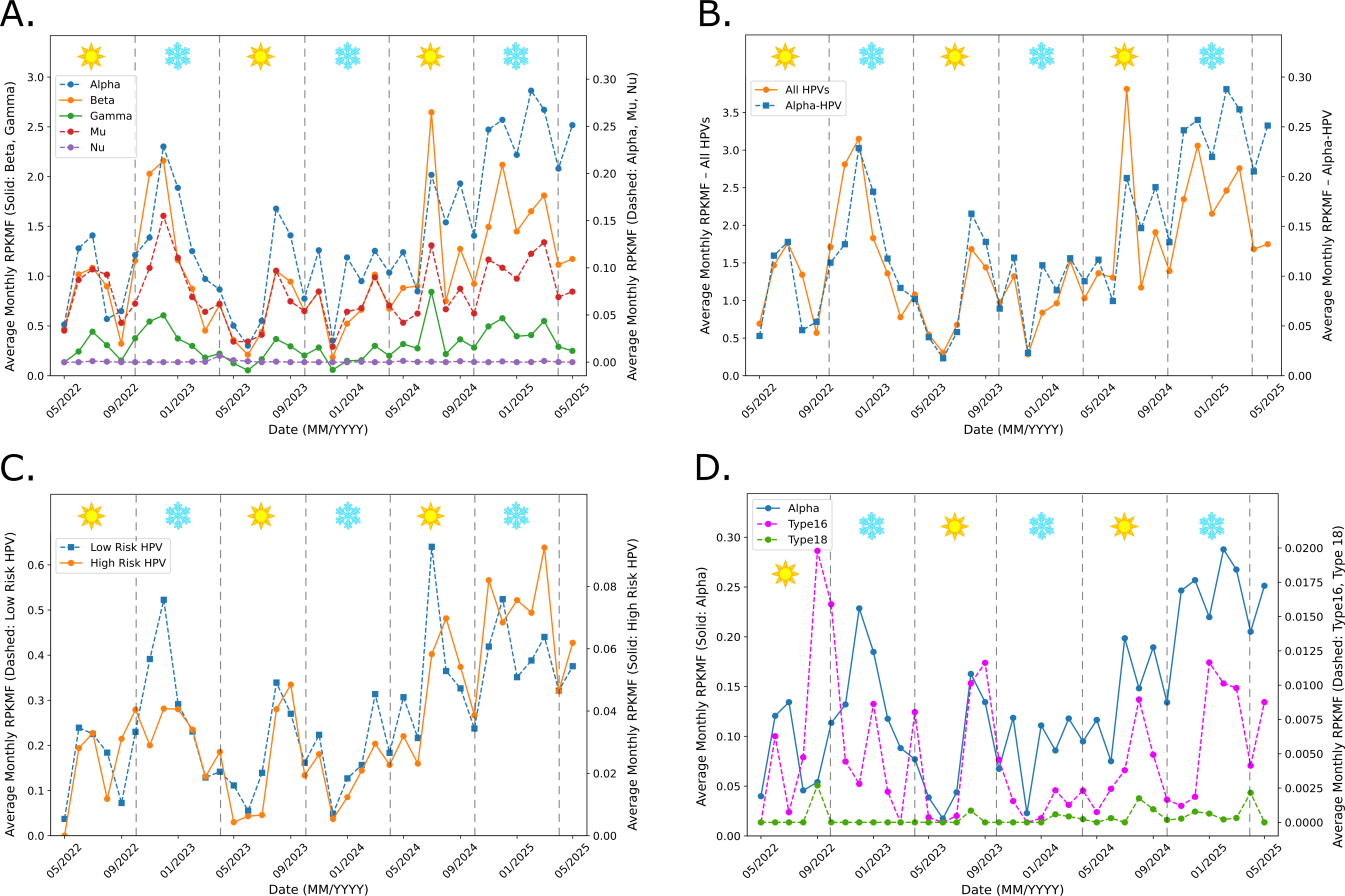
HPV genus-specific abundance. (**A**) Prevalence of alpha-, beta-, gamma-, mu-, and nu- HPV genera measured as average RPKMF from May 2022 to May 2025. (**B**) Comparison of highly oncogenic alpha-HPV against all other HPV genera. (**C**) Comparison of high-risk HPV against low-risk HPV. (**D**) Comparison of alpha-HPV type 16 and alpha-HPV type 18 against all alpha-HPV types. Warmer months are highlighted with a sun icon (May–September). Colder months are highlighted with a snowflake icon (October–April).

We assigned reads classified as HPV to one of five genera: alpha-, beta-, gamma-, mu-, and nu-papillomaviruses based on EsViritu taxonomic labels. During the 3-year sampling period, beta-HPV consistently showed the highest average abundance statewide, followed by gamma-HPV (Fig. 3A). Both genera exhibited semi-seasonal twice-yearly spikes in read abundance, most notably in mid-2022 (May–July), late 2022 (September–December), mid-2023 (June–August), and mid to late 2024 (June–December). Alpha-HPV, including the high-risk types 16 and 18 strains, was present in lower overall levels but demonstrated short-lived peaks that aligned with most of the broader multi-genus spikes, including those in July 2022, August 2023, and July 2024.

Mu- and nu-HPV were detected at very low levels throughout the study period, with nu-HPV appearing only sporadically. These genera contributed minimally to the overall HPV signal.

We also observed an extended alpha-HPV peak in early 2025 that followed a multi-genus increase in late 2024. When comparing alpha-HPV with the total HPV signal across all genera, the two trends generally rose and fell in parallel (Fig. 3B). However, alpha-HPV remained consistently lower in abundance and showed a distinct peak in February 2025, where it peaked roughly 2 months after the winter spike in total HPV signal.

To assess alpha-HPV prevalence, we compared low-risk types (6, 11, 40, 42, 43, 44, 54, 61, 70, 72, 61, and 81) with high-risk types (16, 18, 31, 33, 35, 39, 45, 51, 52, 56, 58, 59, 66, and 68). Interestingly, while the overall abundance of low-risk types was higher than that of high-risk types, similar time-series patterns were observed across 3 years of sampling with minor exceptions (Fig. 3C). This was especially evident as seen in the winter spikes of low-risk types during late 2022 and high-risk types during early 2025. Throughout the sampling durations, our results show that high-risk alpha-HPV types reach all-time high peaks during late 2024 to early 2025, possibly indicating an increase in HPV statewide.

Finally, we looked at the abundance of the alpha-HPV types responsible for causing over 70% of all known cervical cancers, namely HPV16 and 18. Here, we found that type 18 alpha-HPV was present in relatively lower abundances compared to type 16 (Fig. 3D). While both types peaked together during the warmer months across all 3 years of sampling, a concordant pattern of viral prevalence was absent. Type 16 was observed to peak and dip across all sampling years, while type 18 was absent during most months.

### HPV wastewater reads resolve into clinically relevant phylogenetic lineages

To evaluate the diversity and taxonomic resolution of HPV sequences detected in wastewater, we built a maximum-likelihood phylogenetic tree using representative HPV genomes from the PaVE (The Papillomavirus Episteme) database (39). Within this public database, HPV taxonomic classification is represented in the order of genus - > species - > type, and we utilized the same nomenclature in our work. Multiple species of HPV (e.g., Alpha10, Alpha1, etc.) belong to each genus of HPV (e.g., Alpha, Beta, etc.), and each species has its own group of species-specific HPV types (e.g., HPV1REF and HPV2REF, etc.). All 224 species-specific HPV types for each HPV Genus are listed in Table S1 (Sheet 1).

**Fig 4:**
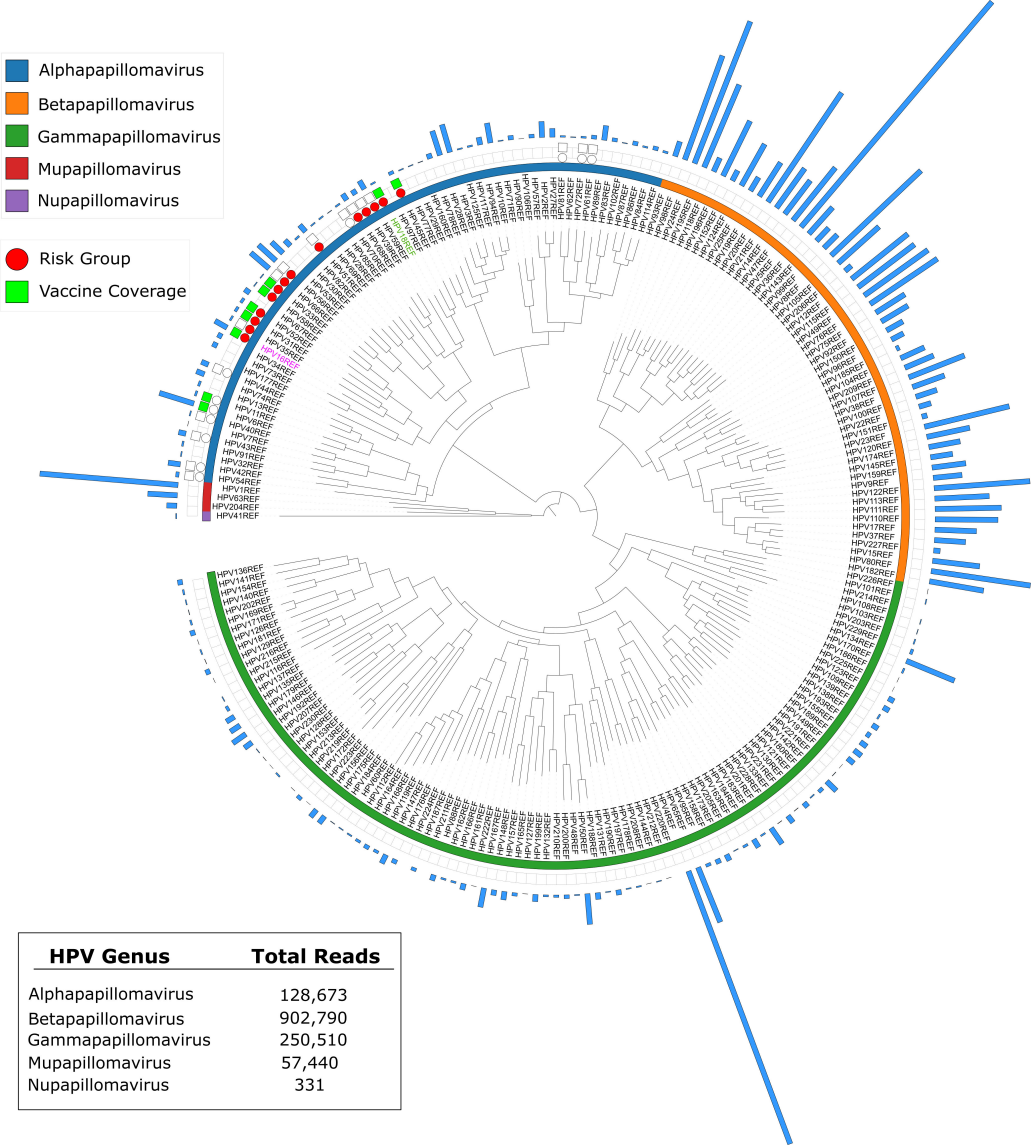
Phylogenetic resolution of wastewater-derived HPV reads. Phylogenetic reconstruction of HPV genera and their types. The outer bar chart represents the number of reads mapping to each type as determined by competitive multimapping. Risk grouping of high-risk alpha-HPV types is highlighted with a red circle, while vaccine coverage is highlighted with a green square. Empty circles and squares indicate low-risk types and zero vaccine coverage, respectively. High-risk alpha-HPV type 16 and alpha-HPV type 18 labels are highlighted in pink and green, respectively.

The phylogenetic tree was built using all 224 species-specific HPV types as representative HPV genomes (39), and each branch tip was colored by genus with bar charts overlaid showing how many reads mapped to each genome (Fig. 4). HPV genomes were also annotated with symbols for risk classification (high-risk and low-risk) and vaccine coverage.

Mapping data confirmed broad representation across alpha-, beta-, and gamma-papillomaviruses. Beta-HPV types showed the widest spread, with low-to-moderate read support across a broad range of genomes. One genome, beta-HPV type 5 (HPV5REF), dominated within this genus, receiving over 95,000 mapped reads. Similarly, in the gamma genus, gamma-HPV type 4 (HPV4REF) had the strongest support with over 93,000 reads (Table S1, Sheets 3–4).

In contrast, alpha-HPV types, which include high-risk mucosal genotypes, showed tighter clustering of mapped reads. Among the alpha-HPV genotypes detected, alpha-HPV type 6 (HPV6REF) and alpha-HPV type 3 (HPV3REF), members of the Alpha10 and Alpha2 species, respectively, accounted for the highest number of mapped reads. These are considered low-risk alpha-HPV types associated with benign mucosal infections. We also recovered several high-risk, cancer-associated genotypes, including alpha-HPV type 16 (HPV16REF) and alpha-HPV type 18 (HPV18REF), the two genotypes responsible for most HPV-associated cancers, which belong to the Alpha9 and Alpha7 HPV species, respectively. These oncogenic types were consistently present across the time series despite their lower abundance (Fig. 3D).

In total, all nine HPV types targeted by the 9-valent vaccine (HPV6, 11, 16, 18, 31, 33, 45, 52, and 58) were identified in our phylogenetic tree. This demonstrates that the hybrid-capture sequencing approach can detect both high-risk and low-risk HPV types from complex environmental samples, including vaccine-preventable lineages (Fig. 3C), and may suggest that wastewater tracking can be used to indirectly report on periods of low vaccination if the signal picks up. Alpha-HPV genotypes, including high-risk types, were reliably recovered, which supports the ability of hybrid-capture sequencing to resolve clinically relevant alpha-HPV types.

### HPV genomes achieve near-complete coverage with hybrid capture sequencing

**Fig 5:**
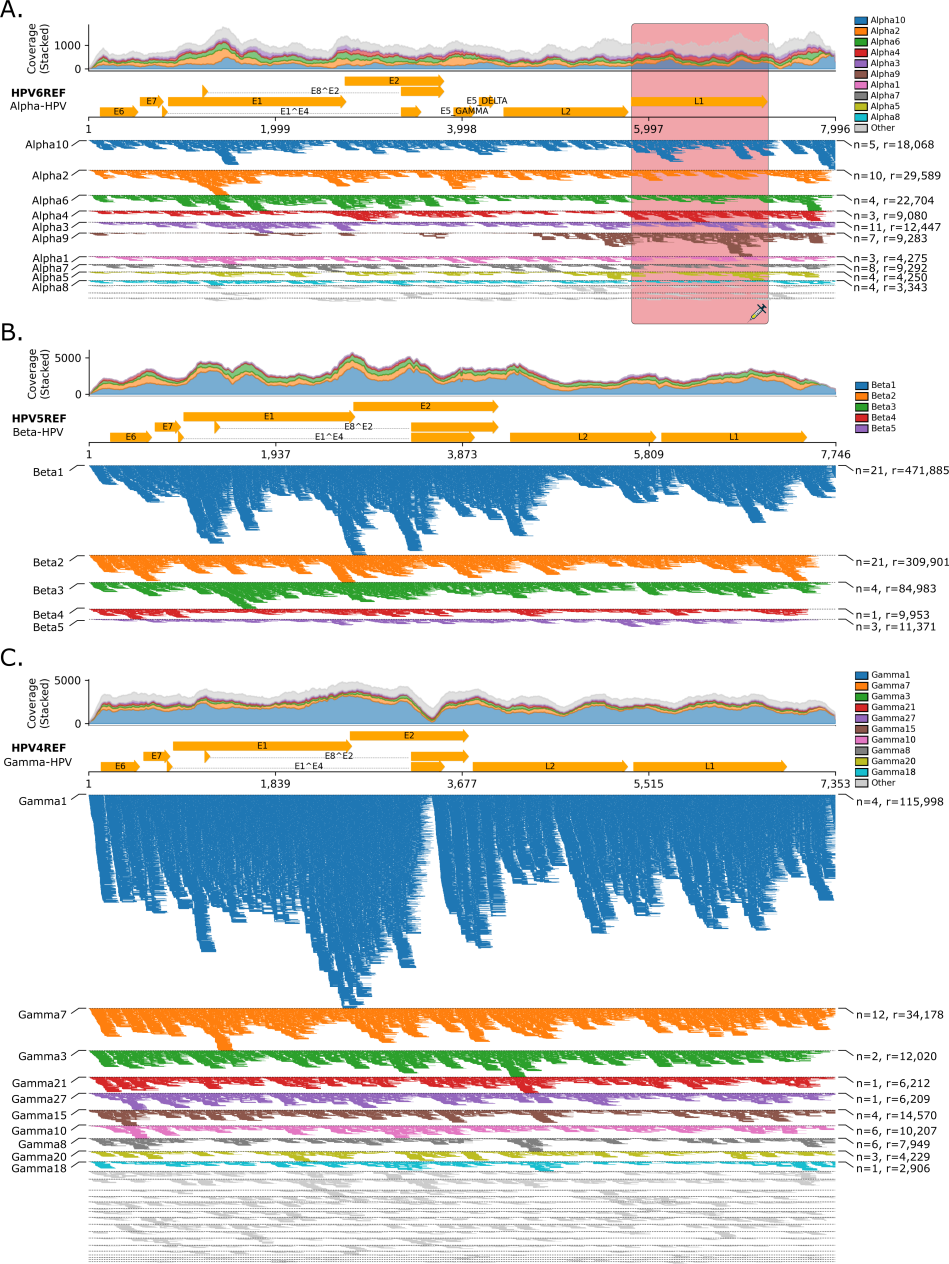
Genome-wide coverage of HPV genera from wastewater using hybrid-capture sequencing. Multi-stack coverage maps of alpha-, beta-, and gamma-HPV reads mapped to their respective genus-level species types. Top 10 species (left) are highlighted with non-gray colors, while the remaining species are highlighted in gray. Stacked coverage map (top) shows coverage of reads mapped to the top 10 species. Number of types per genus-level species (N) and the number of reads mapped (R) are also shown (right). Vaccine target regions on the genome are highlighted in red with a syringe icon. (**A**) Alpha-HPV reads are mapped to all alpha-HPV species. (**B**) Beta-HPV reads are mapped to all beta-HPV species. (**C**) Gamma-HPV reads are mapped to all gamma-HPV species.

To visualize genome-wide coverage within each HPV genus, we aligned all available genomes per genus using MAFFT (40) and selected the genome within the genus with the highest read count to act as an anchor. Each genome represents a single HPV type, belonging to its specific HPV species within the same genus. Classified reads from all members of a given genus were then transposed to this anchor genome coordinate space to generate read stack plots and coverage maps, allowing for intra- and inter-genus comparisons of sequencing depth and coverage breadth (Fig. 5).

In Fig. 5A, we show the alpha-HPV multi-coverage map, with alpha-HPV reads transposed onto the HPV type 6 (HPV6REF) reference genome. Here, the HPV type 6 reference genome, one of the five HPV types belonging to the Alpha10 HPV species, was selected since it had 11,573 reads mapped to it, the highest across all alpha-HPV types. In Fig. 5A, we highlight the top 10 alpha-HPV species by read count. For instance, five unique alpha HPV types belong to the Alpha10 species, highlighted with "n" to the right of each coverage map in the figure (Table S1, Sheet 2). Together, these five types account for 18,068 alpha-HPV reads, denoted by “r,” which map to various regions across the alpha HPV anchor genome. The same coverage map was replicated for the remaining alpha-HPV species, with the top 10 presented in a non-gray color, and the rest in gray. We found that the Alpha10, Alpha2, and Alpha6 species together accounted for a majority of alpha-HPV reads, within which HPV types 6, 3, and 66, respectively, possessed the most reads mapped among the types within a species (Table S1, Sheet 2). Read stacks were primarily concentrated around two low-risk mucosal types, HPV type 6 and HPV type 3 (HPV3REF), which together accounted for most alpha-HPV-classified reads (Fig. 4). Coverage across the genome was variable, but key regions such as the L1 and L2 capsid genes and E6/7 oncogenes showed consistent detection signals across multiple alpha-HPV species, confirming that hybrid-capture sequencing recovers clinically relevant alpha-HPV regions from wastewater (41).

Similarly, Fig. 5B shows beta-papillomavirus coverage using HPV type 5 (HPV5REF) as the anchor genome. This genus had the broadest read support across types, with over 900,000 mapped reads. Among these was HPV type 5, one of the 21 known HPV types from the Beta1 species, which contributed the largest fraction of reads (95,151 reads). Taking together, all 21 types belonging to Beta1, 471,885 reads were found to originate from this HPV species (Table S1, Sheet 3). Coverage was even across most of the genome for all beta-HPV species, indicating widespread environmental presence and consistent detection of multiple beta-HPV types.

In Fig. 5C, gamma-HPV reads were transposed to HPV type 4 (HPV4REF). Type 4 HPV is a member of the Gamma1 species along with three other gamma-HPV types (Types 65, 95, and 173). Type 4 HPV had the highest read support, with over 93,000 classified reads (Table S1, Sheet 4). Like beta, the gamma-HPV signal was dominated by a single high-abundance type belonging to the Gamma1 species but also showed higher baseline support with broad and relatively uniform coverage across all types.

## DISCUSSION

Our work showcases the ability to detect and track all known oncogenic viruses in wastewater samples across vast geographic regions using a sequencing-based metagenomic approach. Building on previous WBE studies, we demonstrate the utility of a hybrid-capture technique to detect oncogenic viruses from wastewater, classifying and characterizing them at the genus level. Using similar methods previously applied to track viruses such as mpox and avian influenza (32–34), we now extended this analysis to HPV, hepatitis viruses, BKV, and more. Here, we showcase how hybrid-capture sequencing can greatly enhance and complement traditional WBE through longitudinal time-series plots, genus-level taxonomic classifications, and phylogenetic reconstruction with species- and type-level diversity.

Over 3 years of sequencing, we characterized nearly 9,000,000 viral reads into nine distinct oncogenic viruses, as well as all known hepatitis viruses, measuring their relative abundance and identifying genomic regions from which the reads originated. Although relative abundance cannot be used as a replacement for absolute abundance when tracking viral prevalence, our supplementary analysis standardizing samples with an SV40 spike-in showed similar prevalence trends. While SV40 spike-ins were only added starting September 2023, the results showcase how both raw and normalized read abundances for several key viruses share similar temporal patterns. Thus, the overall conclusion of rising viral prevalence remains consistent (Fig. S3).

By combining changes in viral signal over time with changes within the viral genome, we can track current clinically relevant strains and sub-strains, while maintaining an overview of all oncogenic viruses across the geographic landscape. Such information is valuable for risk assessment and for developing public health policies that inform officials about the degree of risk in their communities. Tracking the evolution of high-risk viral strains at the molecular level can also guide the design of new vaccines, the identification of novel drug targets, and the development of diagnostic tools.

Our results validated the presence of all oncogenic viruses in wastewater from Texas using hybrid-capture sequencing, consistent with previous studies which utilized targeted approaches to detect viruses such as BKV, HPyV, MCV, and HPV in wastewater (28, 42–44). While these studies were carried out in different geographical locations, the key conclusion remains the same: that the genetic material of oncogenic viruses circulates in wastewater and can be detected using appropriate WBE methods. After 2024, we observed peak relative abundance for several oncogenic viruses, including HTLV-1, HPV, HCV, KSHV, MCPyV, and BKV. Notably, viral loads increased sharply across all targets during the summer months of 2023 and again in 2024. While no consistent annual seasonal cycle was evident, these summer spikes may reflect increased travel and interpersonal contact during vacation and holiday periods in academic settings such as colleges. The removal of pandemic-related social distancing could also have contributed to higher transmission of sexually transmitted oncogenic viruses such as HPV and likely underlies the sharp spike observed in late 2022 that coincided with post-COVID mixing and the immunity debt period documented for respiratory viruses such as the Respiratory Syncytial Virus (RSV) (45). While the relative abundance of viral reads varied across viruses, our hybrid-capture method was successful in detecting even low amounts of virus. These include HTLV-1 and KSHV, which were detected at disproportionately low levels relative to other viruses. Interestingly, previous studies also showed very low levels of KSHV in wastewater, hypothesizing that this could be due to the non-enteric nature of the virus (46). However, low detection levels could also reflect low prevalence of viral load in wastewater or reduced capture efficiency of the hybrid-capture probes.

With our next objective to identify genomic regions from which viral reads originated, we mapped sequenced reads to their corresponding reference sequences. The mapping step of our analysis was carried out under some key assumptions. These include carrying out a read assignment using a multimapping strategy, where reads that map equally well to multiple references with our mapping tool (minimap2) are assigned to all references with the best alignment score. In the case of hepatitis viruses, the reference genome with the highest total mapped reads was selected as the representative reference genome. Here, we assume the reference with the most mapped reads best represents the dominant circulating hepatitis strain, although minor strains may be underrepresented.

After mapping classified reads to their respective reference genomes, we found that among the five hepatitis viruses (Fig. 2; Fig. S1), near-complete genomic coverage of HAV and HBV reads was observed, with semi-complete coverage obtained for HEV. In contrast, HCV and HDV coverage was highly fragmented, with nearly 95% of reads mapping to a short segment in the NS1/NS2 region for HCV, and almost 90% of reads mapping to segments in the LHD region for HDV. The NS2 region on the HCV genome encodes a nonstructural protein, highly involved in the replication and production of new HCV particles, and its presence in wastewater samples could be used as a proxy to assess the spread of HCV (47). The LHD region in HDV is known to be a key factor in HDV RNA packaging with the HBV envelope protein (48). Since HDV can only co-infect an individual with HBV, the presence of HDV reads can be correlated with HBV reads at the site level, facilitating a better understanding of HDV viral prevalence in a community as opposed to HBV alone. We also found that multiple HAV reads and a smaller number of HBV reads mapped to vaccine target regions such as the capsid protein and surface antigen protein regions, respectively, indicating that hybrid-capture sequencing can recover sequences relevant for monitoring potential resistance mutations or immune escape.

To further assess taxonomic resolution obtained by this method, we focused on HPV due to its medical relevance, multiple genera, and varying health risks among subtypes. Across 3 years, beta- and gamma- HPV showed higher abundance than alpha-, mu-, and nu-HPV, likely reflecting more efficient cutaneous transmission of beta and gamma types compared to sexual transmission routes of alpha types. These results correlate with other studies studying HPV prevalence in wastewater, where relatively higher abundances of beta-HPVs were detected along with high-risk alpha-HPVs (28, 49). Although less abundant, alpha-HPVs are clinically significant because many types are high-risk mucosal genotypes linked to cancer in humans.

Comparing alpha-HPVs to all HPV genera showed lower overall abundance but similar timing of peaks and dips. This pattern suggests that HPV prevalence across sites is not driven by a single genus, with viral abundance in multiple genera tending to rise and fall together. This is significant because monitoring these parallel trends could help identify broader shifts in pathogenic HPV transmission within the community.

When comparing high-risk and low-risk alpha-HPV types, high-risk variants were consistently less abundant. This pattern may reflect the impact of vaccination campaigns targeting high-risk types, which could lower their prevalence in wastewater. In contrast, low-risk types, which are not typically vaccine targets and pose less severe health risks, were detected in higher abundances across the state (Fig. 3C). Thus, this approach may help report on vaccine uptake. However, when assessing HPV data, it must be noted that HPV vaccination completion records in Texas are very low, with the state ranking 48th in the United States (50). Although this reflects the state’s poor vaccination rates, it also provides an ideal setting for future studies to assess the correlation between vaccination rates and viral prevalence, as well as look at public health measures to reduce HPV prevalence.

Together, HPV16 and HPV18 cause more than 70% of all cervical cancer incidences, individually responsible for nearly 55%–60% and 10%–15% of all cases, respectively (51). Our wastewater data showed HPV16 in higher abundance than HPV18, mirroring studies conducted worldwide, where this high-risk HPV type was regularly detected in wastewater (52, 53). HPV16 trends generally mirrored those of all alpha-HPV types, whereas HPV18 remained low except for small peaks in summer months. These differences may reflect variation in viral shedding, persistence, transmissibility, or probe detection sensitivity. Correlating wastewater trends with clinical data will be important to determine whether the lower HPV18 signal reflects true prevalence or under-detection.

Finally, mapping of all HPV reads to their associated genus showcased the ability of hybrid-capture sequencing in resolving viral sequences to the genus- and type-taxonomic levels. For HPV type-level analysis, closely related types within a single HPV species share genomic regions (regions within the conserved L1 capsid gene region), implying that few reads could be assigned to multiple HPV types together. We acknowledge that this is a conservative approach for genus-level detection, and type-level read counts must be interpreted with caution. Our results highlighted the resolution of over 1.3 million HPV reads, with beta-HPV reads comprising the majority, with nearly 900,000 classified reads. Interestingly, HPV type 6 and HPV type 3, low-risk HPV types associated with benign lesions, genital warts, and flat warts, accounted for the most alpha-HPV reads. Their relatively higher prevalence in wastewater could be a consequence of shedding infected skin cells into wastewater during routine bathing or washing, with studies showing HPV detection in domestic bathing water samples (6).

Although hybrid-capture recovered near-complete genomes for many oncogenic viruses, coverage varied across targets, suggesting that genome recovery from wastewater may depend on factors such as viral persistence, transmissibility, and shedding dynamics (and nucleic acid region stability), and that not all oncogenic viruses are equally amenable to broad genome reconstruction. As highlighted earlier, this is visually evident in the case of HCV and HDV, where most of the sequenced reads map to the NS1/NS2 and LHD genomic regions on the reference genome, respectively (Fig. 2B and C). Nonetheless, knowledge of the primary genomic region of origination of viral reads can give valuable information about viral transmission and shedding dynamics.

While our work showcased how hybrid-capture sequencing can successfully assist oncogenic virus detection in wastewater, limitations exist. Poor hybrid-capture probe sensitivity can lead to under-representation of viral targets, convoluting the interpretation of viral prevalences in the process. We have also limited our study by looking at a high-level overview of oncogenic virus prevalence in Texas by taking the sequencing data from all sampling sites together. A key limitation of our study, however, is the relative nature of measured viral abundance. RPKMF in its current form is not an absolute value and can confound results in the context of quantifying viral prevalence in a community. While using a known volume of spike-in allows absolute abundance measurement, our samples only included SV40 spike-ins starting from September 2023. To see the impact of normalization, we compared raw RPKMF with SV40-normalized RPKMF and found similar temporal trends. This shows that the trend of increasing viral prevalence is strong, although RPKMF cannot be interpreted as absolute abundance.

One way to overcome some general limitations in future higher-resolution work is to utilize targeted WBE approaches, such as qPCR/dPCR, parallel to hybrid-capture sequencing to give a more accurate representation of viral abundance in sampling locations over time. However, although these targeted approaches are comparatively cheaper to process and analyze, they lack the ability to resolve viral detections at the genus- and type-level, as well as readily detect novel variants. In contrast, hybrid-capture sequencing allows identification of viral reads at deeper taxonomic levels along with simultaneous monitoring of thousands of viral targets. Thus, taken together, these approaches leverage complementary strengths, offering a robust framework for viral monitoring.

Future work will include site-specific time-series analyses to estimate the risk level at the city/site level. Since the prevalence of high-risk and low-risk HPV may vary at the site level, assessing these data would be crucial to ensure that sites at low risk are not misclassified as high risk simply due to the detection of HPV as a whole at that location. In addition, integration of wastewater signals with vaccination data to evaluate how immunization campaigns influence the prevalence of high-risk viruses such as HBV and HPV can be carried out. To obtain insights into the evolution of high-risk strains and allow for early identification of mutations that enhance viral fitness or reduce vaccine efficacy, variant analyses of viral genes may also provide valuable insight.

In conclusion, this study demonstrates how hybrid-capture sequencing enables consistent detection and genomic characterization of oncogenic viruses in wastewater across large geographic regions. The approach provides sufficient resolution to monitor viral genera and subtypes, including high-risk lineages, and to assess genomic variation relevant to transmission and resistance. To our knowledge, this work is the first to use hybrid-capture sequencing to successfully detect all known oncogenic viruses in wastewater samples. As wastewater-based epidemiology expands, these findings highlight the advancing potential of sequencing-based methods, which can be complemented with targeted approaches to support long-term surveillance of cancer-associated viruses and inform public health strategies.

## MATERIALS AND METHODS

### Sample collection and shipping

Wastewater samples from over 40 sites across 16 cities in Texas were collected, shipped, and processed using methods described by Tisza et al. (34, 36).

Specifically, 100–500 mL of raw wastewater was collected and stored in 500 mL prelabeled sample bottles. Collection sites were coded upon the request of public health officials to mask the identity of the cities where sampling was carried out.

To decontaminate the surface of the collection bottles, 10% bleach was used, followed by sealing samples into biohazard bags within shipping boxes containing ice packs and absorbent pads. The boxes were then shipped to the Alkek Centre for Metagenomics and Microbiome Research at Baylor College of Medicine, Houston, Texas. Upon arrival, samples were barcoded and stored at 4°C prior to processing.

Processing of samples was carried out by first decanting and centrifuging 50 mL of wastewater at 3,374 × *g* for 10 min to separate the solid and liquid fractions. The resulting supernatant was next vacuum-filtered using an ion-based cellulose filter paper, and the now virus-containing filter was put into a bead-beating tube along with lysis buffer. The tube was then run on a homogenizer for 1 min at 5 m/s, rested for 1 min, and run again for 1 min at 5 m/s. After bead beating, samples were centrifuged at 14–17 × 1,000 RPM for 2 min. Once centrifuged, the DNA and RNA were extracted using the Qiagen QIAamp VIRAL RNA Mini Kit.

### Sample processing and sequencing

Reverse transcription of RNA to cDNA was first carried out with the Protoscript II First Strand cDNA Synthesis Kit (New England Biolabs Inc.), NEBNext Ultra II Non-Directional RNA Second Strand Module (New England Biolabs Inc.), and Random Primer 6 (New England Biolabs Inc.) before mixing it with DNA for library construction. Library construction was carried out with Twist Library Preparation EF 2.0 Kit and Twist Universal Adaptor System (Twist Biosciences) using 25 ng of cDNA and DNA mix. Libraries were then pooled with 16 samples, totaling 1,500 ng per pool. Hybridization of probes was carried out with the Twist Comprehensive Viral Research Panel (Twist Biosciences) at 70°C for 16 h. The post-capture pools were further amplified with 12 PCR cycles, and the final libraries were sequenced on the Illumina NovaSeq 6000 SP flow cell to generate 2 × 150 bp paired-end reads, producing raw data files in the binary base call (BCL) format. The raw BCL files were converted to FASTQ format and demultiplexed based on the dual-index barcodes using Illumina bcl2fastq software.

### Sample-level quality control and inclusion criteria

Prior to data aggregation, individual samples were assessed for sequencing adequacy. Over the 3-year study period (May 2022–May 2025), a total of 3,140 samples were taken.

The number of actively sampled sites increased over the study period, beginning with 2–10 sites in May/June 2022 and expanding to over 40 sites by May 2025. To account for this, abundance values were averaged across all contributing sites within a given month rather than summed, ensuring that the addition of new sites did not artificially inflate aggregate RPKMF values.

No additional batch correction was applied across sequencing runs, as all samples within a given pool were processed using the same library preparation and hybridization protocol. Beginning in September 2023, simian virus 40 (SV40) was spiked into all samples at a fixed concentration prior to nucleic acid extraction (see below), providing a run-level internal control for extraction efficiency and sequencing consistency.

### Internal spike-in standardization with SV40

Beginning in September 2023, simian virus 40 (SV40) was added as an exogenous process control to all wastewater samples prior to nucleic acid extraction, providing a known internal standard that undergoes the same extraction, library preparation, hybridization, and sequencing steps as endogenous viral targets.

SV40 reads were identified and quantified using the same EsViritu classification pipeline applied to all other viral targets. To compute a standardized abundance metric, the RPKMF of each target virus in a given sample was divided by the RPKMF of SV40 in that same sample, yielding a ratio proportional to absolute viral concentration. This ratio accounts for sample-to-sample variation in nucleic acid recovery, library preparation efficiency, and sequencing depth, as these factors affect SV40 and endogenous viruses equally.

Standardized abundance ratios were computed for all samples collected between September 2023 and May 2025 and were aggregated monthly using the same TREx pipeline described below. The resulting SV40-standardized time-series trends are presented alongside the original RPKMF-based trends in Fig. S3 to assess concordance between the two metrics. Because SV40 was not introduced until September 2023, standardized values are not available for the first 16 months of the study (May 2022–August 2023). However, the concordance of both metrics during the overlapping 21-month window supports the reliability of RPKMF as a relative measure for the earlier period as well.

### Oncogenic virus sequence assignment with EsViritu

Demultiplexed FASTQ files were processed with BBDuk (v38.84) to carry out adapter removal, quality trimming (Q25), and PhiX read filtering. Reads with a minimum average Phred quality score of < 23 and read length < 50 bp post-trimming were discarded. Removal of reads mapping to PhiX Illumina spike-in or the human reference genome (GCF_000001405.39) was carried out with BBMap (54). Next, the remaining processed reads were classified using EsViritu (v0.2.3) (36) with default settings. Here, the processed reads were aligned to the complete Virus Pathogen Database (v2.0.2) (36) using minimap2 (55), keeping alignments with at least 90% average identity across 90% of the read length with CoverM (https://github.com/wwood/CoverM).

Those virus genomes or reads covering a minimum of either 1,000 nt or 50% of the genome/segment length were considered preliminary detections. Since de-replicated virus sequences can have highly similar genomic regions, consensus sequences from the preliminary detections were extracted using samtools (v1.21) consensus (56). Preliminary consensus sequences were compared pairwise to each other using anicalc (57), and clusters were made from sequences with at least 98% identity with aniclust (57). The longest sequence within the cluster was taken as the final sequence. The sequenced fastq reads were next aligned to a smaller database with references corresponding to the final sequences using the same parameters with minimap2 and CoverM. The virus genomes or read segments with at least 1,000 nt or 50% of the genome length were taken as the final detections.

At low levels in wastewater, the proportion of reads aligning to virus genomes is presumed to correspond to the proportion of viral nucleic acid molecules over the remaining molecules in the sample (36). Thus, we use RPKMF, calculated as Reads Per Kilobase of reference genome/Million reads passing Filtering, as our abundance metric. This is a relative abundance metric, and not an absolute metric for quantification of specific viral particles in a sample.

Per sample abundance, along with coverage metrics and taxonomic profiles, is tabulated and merged into a single table per pool for downstream processing.

### Taxonomic filtering and abundance estimation using TREx

All accession IDs of known oncogenic viruses were collected from the Virus Pathogen Database (v2.0.2) (Table S2). TREx (Taxonomic-based Relative-Abundance Extractor (v0.1); https://github.com/TAILOR-Lab/TREx) was then used to filter EsViritu output tables, retaining only entries matching oncogenic virus accession numbers or taxonomic IDs. Once filtered, TREx combines each viral detection with its associated site-level metadata, including anonymized site identifier and collection date.

For this study, we chose to assess statewide oncogenic virus dynamics on a monthly timeframe. Abundance estimation with TREx was carried out with default settings, with “—sensitive” mode enabled and average RPKMF computation set to “—RPKMF_avg all, month.” Under this configuration, TREx computes the arithmetic mean of per-sample RPKMF values for each virus across all samples collected in a given calendar month, regardless of sampling site. This averaging approach was chosen to provide a statewide overview of viral dynamics suitable for the broad surveillance objectives of this study.

We note that this pooling strategy intentionally masks site-level heterogeneity in viral abundance. Because our objective was to characterize statewide temporal trends rather than spatial variation, we deferred site-specific analyses to planned follow-up work (see Discussion). The monthly averaged RPKMF values generated by TREx for all oncogenic viruses are provided in Supp. Table 3, along with the number of contributing sites and samples per month in Table S4.

### Time-series analysis of the average RPKMF of oncogenic viruses

The average RPKMF values calculated with TREx (v0.1) and aggregated monthly across all collection sites were used as input to plot time-series charts for each oncogenic virus using a custom Python (v3.12.7) script with the matplotlib (v3.9.1) (58) and pandas (v2.3.0) (59) packages (Table S3). Time-series charts for all viruses were plotted on individual plots as a panel (Fig. 3; Fig. S1). Additional graphical illustrations were added to the charts with Inkscape (v1.3.2).

### Mapping of reads to hepatitis A, B, C, D, and E references

Reads classified as hepatitis A, B, C, D, and E were mapped against all their corresponding available reference genomes in EsViritu’s Virus Pathogen Database (v2.0.2) to identify the reference with the highest read support. For instance, this database has two references for HAV (Table S2), and the reference to which more HAV reads mapped was selected for coverage analysis. Thus, the references with the highest read support (HAV: LC373510, HBV: AB625342, HCV: NC_004102, HDV: AM779592, and HEV: LC055972) for each virus were selected for downstream analysis. Coverage was calculated as per-base depth across the full genome, and visualizations included both read depth histograms and feature annotations from corresponding GenBank files. Custom Python scripts using pysam (v0.22.1), Biopython (v1.85) (60), and matplotlib (v3.9.1) were used to generate genome-wide coverage plots (Fig. 2; Fig. S2), with coding regions, including vaccine targets, highlighted for context with Inkscape (v1.3.2).

### HPV genome coverage analysis

EsViritu-classified HPV reads were first mapped using a multimapping strategy to the PaVE database (39) in Geneious Prime (v2025.1.3). For each HPV genus, we built reference multiple sequence alignments with MAFFT (v7.505) using representative genomes from the PaVE database and selected a single anchor genome per genus. We then generated position lift maps from each reference to its genus anchor using the MAFFT alignments as a coordinate guide and lifted sample BAMs to the common anchor coordinate space with pysam while preserving read-group tags for HPV type origin. Per-base depth on the anchor was computed from the lifted BAMs, and coverage summaries were exported as TSV and JSON files. Plots show three alignment panels: coverage across the full anchor genome, GenBank-derived features, and read stacks colored by the most contributing types.

### HPV phylogenetic analysis

Representative HPV genomes were aligned with MAFFT (v7.505), and phylogenetic trees were constructed with IQ-TREE (v2.1.4) (61). Mapped wastewater reads were then used to generate bar graph annotations for each genome, indicating the number of reads mapped. Genus-level rings were also created as annotations for visualization in iTOL (v6) (62). This approach enabled us to place HPV reads within a reference phylogeny; highlight taxonomic breadth across alpha-, beta-, and gamma-papillomaviruses; and annotate clinically relevant types by risk classification and vaccine status.

## Data Availability

Read abundance tables with percent identities of mapping, along with site-specific read counts and taxonomic information, have been included as supplemental material (Tables S3 and S4). Raw sequencing read data are available in the NCBI BioProject database under accession number PRJNA966185. All core code used in this study, including the TREx abundance extraction tool (https://github.com/TAILOR-Lab/TREx) and the EsViritu viral mapping pipeline (https://github.com/cmmr/EsViritu), is publicly available. Custom code developed for read classification, mapping to reference genomes, and generation of time-series figures is available via a dedicated GitHub repository (https://github.com/TAILOR-Lab/oncogenic-viruses-wastewater-study).

## SUPPLEMENTAL MATERIAL

Fig. S1 aem.00547-26-s0001.tiff Abundance of different hepatitis virus reads over 3 years of sampling across Texas.

Fig. S2 aem.00547-26-s0002.tiff Genome-wide read coverage of sampled hepatitis viruses via hybrid-capture sequencing.

Fig. S3 aem.00547-26-s0003.tiff Raw vs. SV40 spike-in normalized RPKMF of all 12 viruses over 3 years of sampling.

Supplemental legends aem.00547-26-s0004.docx Descriptive legends for supplemental material.

Table S1 aem.00547-26-s0005.xlsx HPV genera with PAVE database nomenclature, with the number of reads mapping to alpha, beta, gamma, mu, and nu HPV types.

Table S2 aem.00547-26-s0006.xlsx Accession IDs used to extract oncogenic virus-specific RPKMF data from EsViritu outputs with TREx.

Table S3 aem.00547-26-s0007.xlsx Average monthly RPKMF of all 12 tested viruses from May 2022 to May 2025.

Table S4 aem.00547-26-s0008.xlsx Site-specific RPKMF of all 12 viruses with date of sampling and anonymized site and city information.

ASM does not own the copyrights to Supplemental Material that may be linked to, or accessed through, an article. The authors have granted ASM a non-exclusive, world-wide license to publish the Supplemental Material files. Please contact the corresponding author directly for reuse.
