# Supplementary figures and images for: Detection, persistence, and rising prevalence of oncogenic viruses revealed by wastewater metagenomics
Source: Appl Environ Microbiol. 2026 May 13;92(6):e00547-26. doi: 10.1128/aem.00547-26 (PMC13274453; doi:10.1128/aem.00547-26)

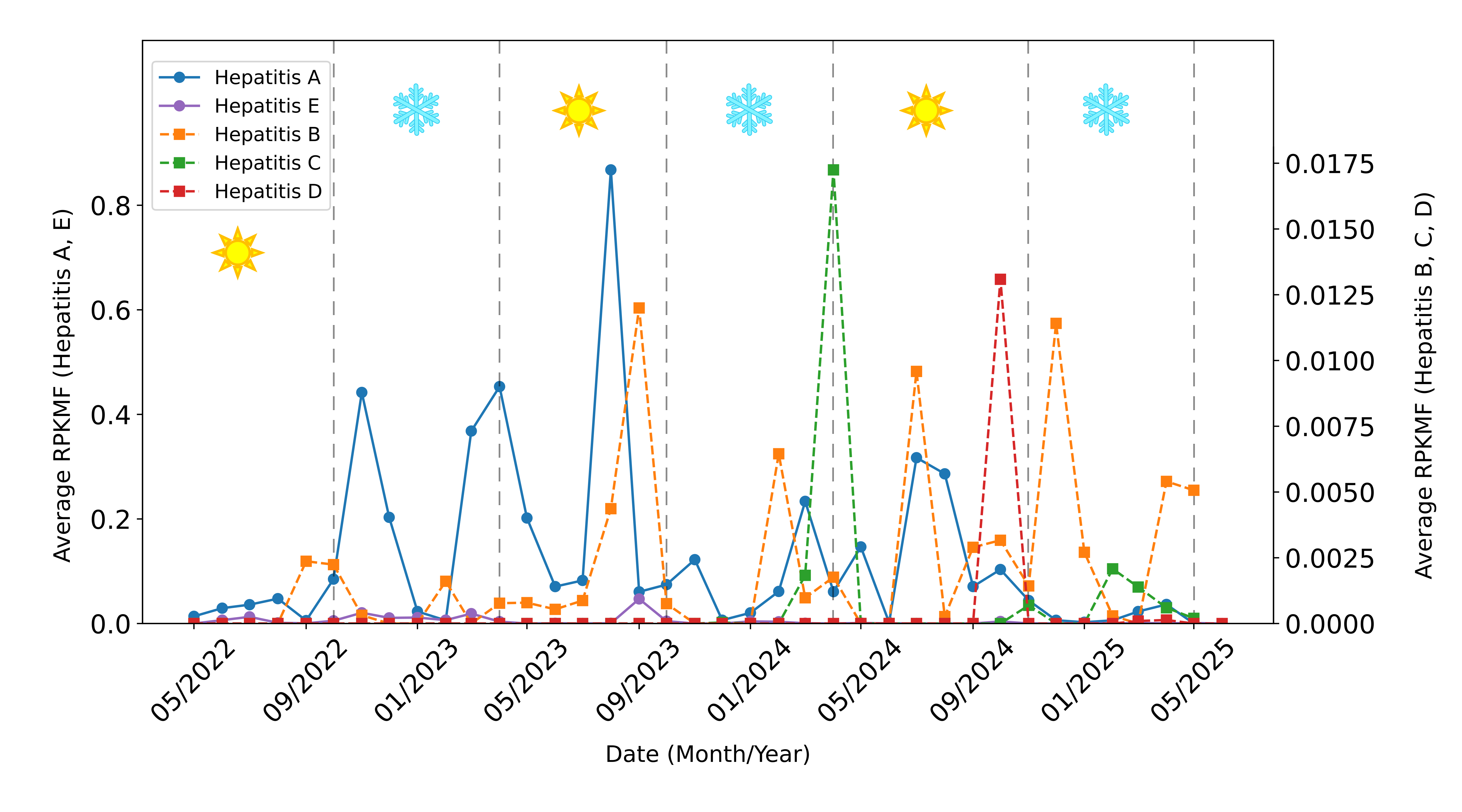

Supplement: Fig. S1 — Abundance of different hepatitis virus reads over 3 years of sampling across Texas. [file aem.00547-26-s0001.tiff]

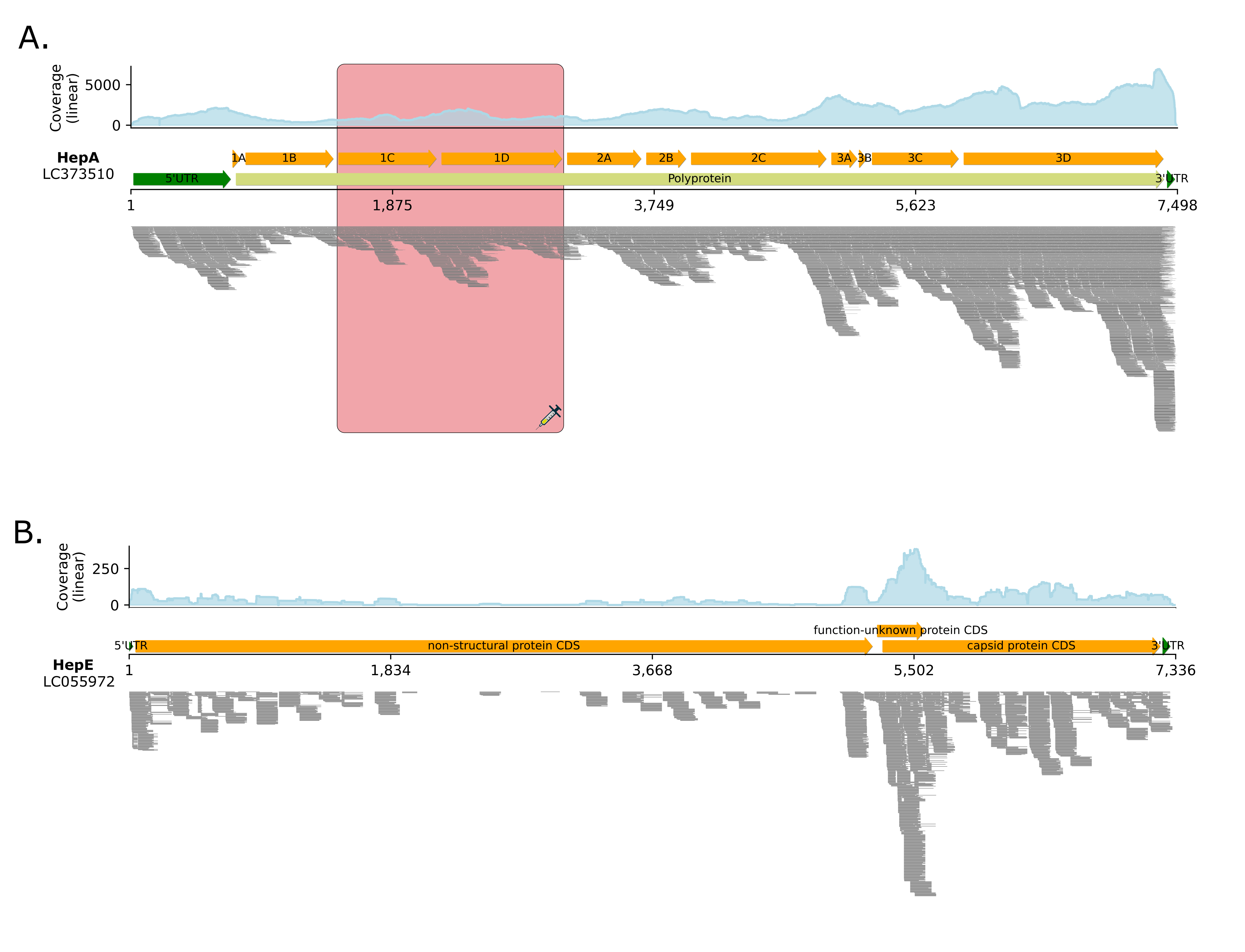

Supplement: Fig. S2 — Genome-wide read coverage of sampled hepatitis viruses via hybrid-capture sequencing. [file aem.00547-26-s0002.tiff]

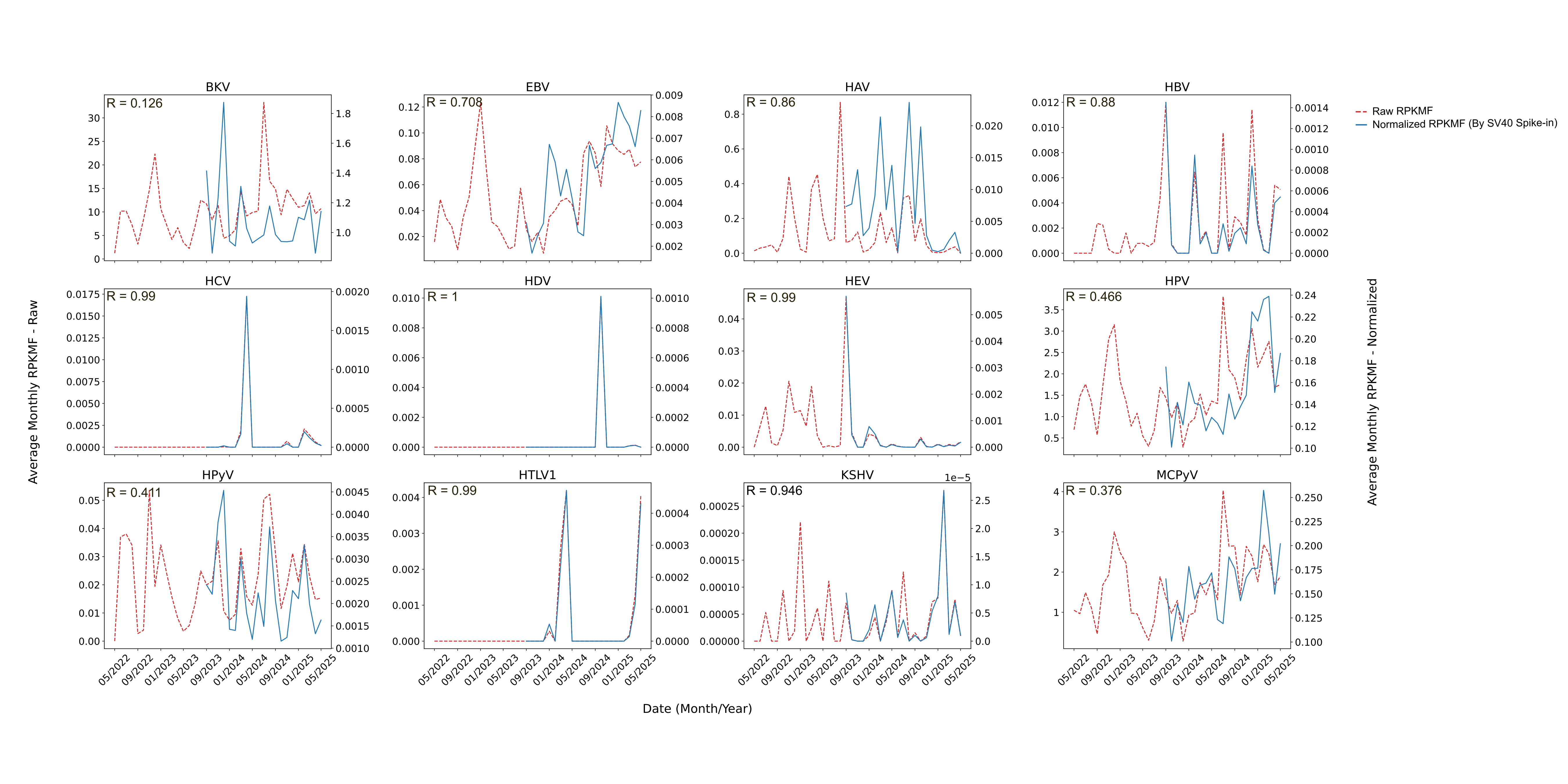

Supplement: Fig. S3 — Raw vs. SV40 spike-in normalized RPKMF of all 12 viruses over 3 years of sampling. [file aem.00547-26-s0003.tiff]
