# Supplementary material for: Detection, persistence, and rising prevalence of oncogenic viruses revealed by wastewater metagenomics
Source: Appl Environ Microbiol. 2026 May 13;92(6):e00547-26. doi: 10.1128/aem.00547-26 (PMC13274453; doi:10.1128/aem.00547-26)
Supplement: Supplemental legends — Descriptive legends for supplemental material. [file aem.00547-26-s0004.docx]

**Title:** Detection, Persistence, and Rising Prevalence of Oncogenic Viruses Revealed by Wastewater Metagenomics

**Authors:** Harihara Prakash M.S^1,2^, Ryan K. Perez B.A^1^, Matt Ross Ph.D^1,3^, Michael Tisza Ph.D^1,3^, Sara J. Javornik Cregeen Ph.D^1,3^, Jennifer Deegan MPAff^4^, Joseph F. Petrosino Ph.D^1^, Eric Boerwinkle Ph.D^#,4^, Justin R. Clark Ph.D^#,1,2^, Anthony W. Maresso Ph.D^#,1,2,3^

**Supplemental Material Legends**

**Supplemental Figure 1: Abundance of Hepatitis Family Virus Reads over Three Years of Sampling Across Texas.** Mean viral signal of Hepatitis A, B, C, D, E viruses measured in average monthly RPKMF over time in samples taken from wastewater and measured using hybrid-capture probes. Warmer months are highlighted with a sun icon (April-September). Colder months are highlighted with a snowflake icon (October-March). Seasonal boundaries were defined using statewide average temperatures to determine the midpoints between the summer and winter temperature extremes. While Hepatitis A, D and E are not oncogenic by themselves, they have been included to understand Hepatitis transmission dynamics broadly.

**Supplemental Figure 2: Genome-Wide Read Coverage of Sampled Hepatitis Viruses via Hybrid-Capture Sequencing.** Histogram (top) shows coverage depth across complete genome. Genomic annotations across the genome are highlighted with arrows (middle). Read distribution shown by stacked reads (bottom). Vaccine target regions on the relevant Hepatitis Genomes are highlighted in red with a syringe icon. (A) Reads mapped to reference Hepatitis A genome (Accession: LC373510). (B) Reads mapped to reference Hepatitis E genome (Accession: LC055972)

**Supplemental Figure 3: Raw vs SV40 Spike-In Normalized RPKMF of All 12 Viruses over Three Years of Sampling.** Time-series plots showcasing temporal dynamics of all 12 viruses used in the study measured as RPKMF. Dotted red line indicates raw average monthly RPKMF and the blue line indicates normalized RPKMF (by SV40 spike-in). SV40 was added as a spike-in control from September 2023 onwards, and Pearson correlation coefficient (R) was calculated between raw and normalized RPKMF from that date. Left-side y-axis measures raw RPKMF while right-side y-axis measures the normalized RPKMF. While relatively lower R values were observed for HPV, MCPyV, HPyV and BKV, the overall trend of rising viral abundance is consistent across both normalized and raw measurements.

**Supplemental Table 1: HPV Genera with PAVE Database Nomenclature with Number of Reads Mapping to Alpha, Beta, Gamma, Mu and Nu HPV Types.**

**Supplemental Table 2: Accession ID's Used to Extract Oncogenic Virus Specific RPKMF Data from EsViritu Outputs with TREx**

**Supplemental Table 3: Average Monthly RPKMF of All 12 Tested Viruses from May 2022-May 2025**

**Supplemental Table 4:** **Site Specific RPKMF of All 12 Viruses with Date of Sampling and anonymized Site and City Information**
